# Hibiscus Collagen Alternative (VC-H1) as an Oral Skin Rejuvenating Agent: A 12-Week Pilot Study

**DOI:** 10.3390/ijms26157291

**Published:** 2025-07-28

**Authors:** Yujin Baek, Ngoc Ha Nguyen, Young In Lee, Min Joo Jung, In Ah Kim, Sung Jun Lee, Hyun Min Kim, Ju Hee Lee

**Affiliations:** 1 Department of Dermatology & Cutaneous Biology Research Institute, Yonsei University College of Medicine, Seoul 03722, Republic of Korea; uj200076@yuhs.ac (Y.B.); ngocha7996@yuhs.ac (N.H.N.); ylee1124@yuhs.ac (Y.I.L.); 2 Department of Dermatology, University of Medicine and Pharmacy at Ho Chi Minh City, Ho Chi Minh City 17000, Vietnam; 3 Scar Laser and Plastic Surgery Center, Yonsei Cancer Hospital, Seoul 03722, Republic of Korea; 4 Global Medical Research Center Co., Ltd., Seoul 06526, Republic of Korea; mjung11@gmrc.co.kr (M.J.J.); inah@gmrc.co.kr (I.A.K.); 5 Liting Plastic Surgery, Seoul 06035, Republic of Korea; justdisj@naver.com; 6 Rawga Inc., Seongnam-si 13590, Republic of Korea; raw@rawga.com

**Keywords:** elasticity improvement, *Hibiscus sabdariffa*, plant-based collagen, skin hydration, skin rejuvenation, wrinkle reduction

## Abstract

Skin aging causes reduced hydration, elasticity, and increased wrinkles. Recent safety and compliance concerns over oral collagen supplements have increased interest in plant-based alternatives like *Hibiscus sabdariffa* with antioxidant and anti-aging properties. However, clinical evidence regarding its efficacy remains limited. We aimed to evaluate the effects of this plant-based collagen alternative (VC-H1, Hibiscus Enzyme Extract) supplement on skin hydration, transepidermal water loss (TEWL), desquamation, elasticity, and wrinkle reduction in photoaged individuals. A randomized, double-blind, placebo-controlled clinical trial was conducted with 98 participants (aged 35–60 years) presenting with dry skin and periorbital wrinkles. Participants randomly received 1.5 g/day of VC-H1 or placebo for 12 weeks. Skin hydration, TEWL, deep moisture, keratin index, elasticity, and wrinkle parameters were assessed at baseline, 6 weeks, and 12 weeks. VC-H1 supplementation significantly increased skin hydration, reduced the TEWL and keratin index, and improved deep moisture content for those receiving it compared with the controls. Wrinkle depth significantly decreased, and skin elasticity also improved. Those in the VC-H1 group showed greater overall improvement than those in the control group. Oral VC-H1 supplementation significantly improved skin hydration, elasticity, and wrinkle reduction, suggesting its potential as a plant-based alternative to traditional collagen supplements for skin rejuvenation.

## 1. Introduction

Skin aging is a common aesthetic concern worldwide, driven by intrinsic and extrinsic factors [1]. These processes stimulate the activity of matrix metalloproteinases (MMPs), including collagenase, elastase, and hyaluronidase, leading to the degradation of collagen, elastin, and hyaluronic acid, which ultimately compromises the skin barrier [2,3,4]. Clinically, the effects manifest as reduced skin hydration, loss of elasticity, wrinkle formation, and abnormal desquamation [1,5].

Oral skin rejuvenation agents, particularly collagen sourced from porcine, bovine, and marine sources, are widely used to enhance skin hydration and elasticity and to reduce wrinkles [6,7]. However, concerns regarding safety, potential contamination, and the unpleasant odor associated with marine collagen can negatively impact patient compliance [6,8,9,10]. There is growing interest in plant-based alternatives, which offer several benefits, including the absence of zoonotic disease risk, more favorable sensory profiles, and additional bioactive properties—such as antioxidant, anti-inflammatory, regenerative, wound-healing, and photoprotective effects [11].

*Hibiscus sabdariffa*, commonly known as roselle, is a medicinal plant with well-documented therapeutic applications in dermatology and general medicine. Preclinical studies have revealed its antioxidant, anti-inflammatory, antimicrobial, wound-healing, and anti-aging properties [12,13,14]. Notably, *H. sabdariffa* extract has been shown to inhibit MMP activity, protect against UV- and pollution-induced oxidative stress, and stimulate collagen synthesis, making it a potential intervention for skin rejuvenation [15,16]. Its rich composition of polyphenols, anthocyanins, and organic acids contributes to its ability to mitigate oxidative damage, enhance skin hydration and elasticity, and improve the overall appearance of the skin [17,18,19]. Additionally, its abundant free amino acid disposal has shown collagen-boosting capabilities, leading to reductions in wrinkles and skin roughness observed in laboratory studies [19,20]. Furthermore, its efficacy is supported by clinical evidence, as it improves hydration, reduces transepidermal water loss (TEWL), modulates sebum secretion, and increases systemic antioxidant levels after 6 months of supplementation [21]. These bodies of evidence potentiate the *H. sabdariffa* extract as a promising plant-based alternative for collagen supplementation in skin rejuvenation.

Despite these promising findings, clinical studies in which *H. sabdariffa* was evaluated as an oral skin rejuvenation agent are still scarce [21]. Therefore, the aim of this study was to assess the effects of oral plant-based collagen alternative (VC-H1, Hibiscus Enzyme Extract) supplementation on photoaged skin over a 12-week period, focusing on skin hydration, desquamation, wrinkle reduction, and improvements in texture and elasticity. The results are expected to provide valuable insights into the potential of VC-H1 as a safe and effective plant-based alternative to animal-derived collagen supplements.

## 2. Results

### 2.1. Participant Characteristics

**Figure 1:**
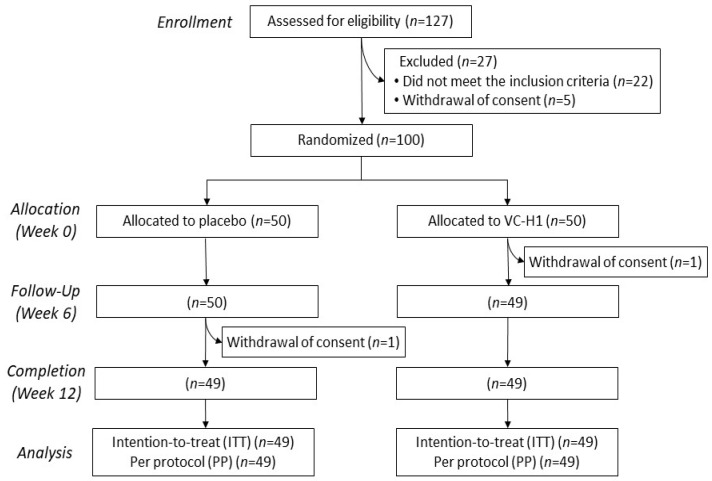
Flowchart of the procedure used to recruit, screen, and randomize the participants.

Overall, 127 participants were screened, and 100 individuals who met the inclusion criteria were randomized into the VC-H1 (n = 50) and control (n = 50) groups. We included 49 participants from each group in the final analysis, all of whom were Koreans (Figure 1). Baseline characteristics are summarized in Table S1. The mean age was 44.6 ± 6.9 years in the control group and 43.4 ± 6.0 years in the VC-H1 group. The baseline skin hydration index was 42.0 ± 4.9 AU (arbitrary unit) in the control group and 43.1 ± 0.6 AU in the VC-H1 group, while the visual assessment score for periorbital wrinkles was 4.3 ± 0.9 in both groups. Therefore, no significant differences in patients’ demographics were observed between groups at baseline, strengthening the validity of the comparative findings. Compliance rates exceeded 98% in the two groups (Table S2), with no significant differences. Dietary intake and physical activity during the intervention period, as shown in Table S3, did not significantly differ. We also did not observe any abnormalities in the hematologic, blood chemistry, or urine tests (Tables S7–S10).

### 2.2. Skin Hydration, TEWL, Skin Moisture, and Keratin Index

**Figure 2:**
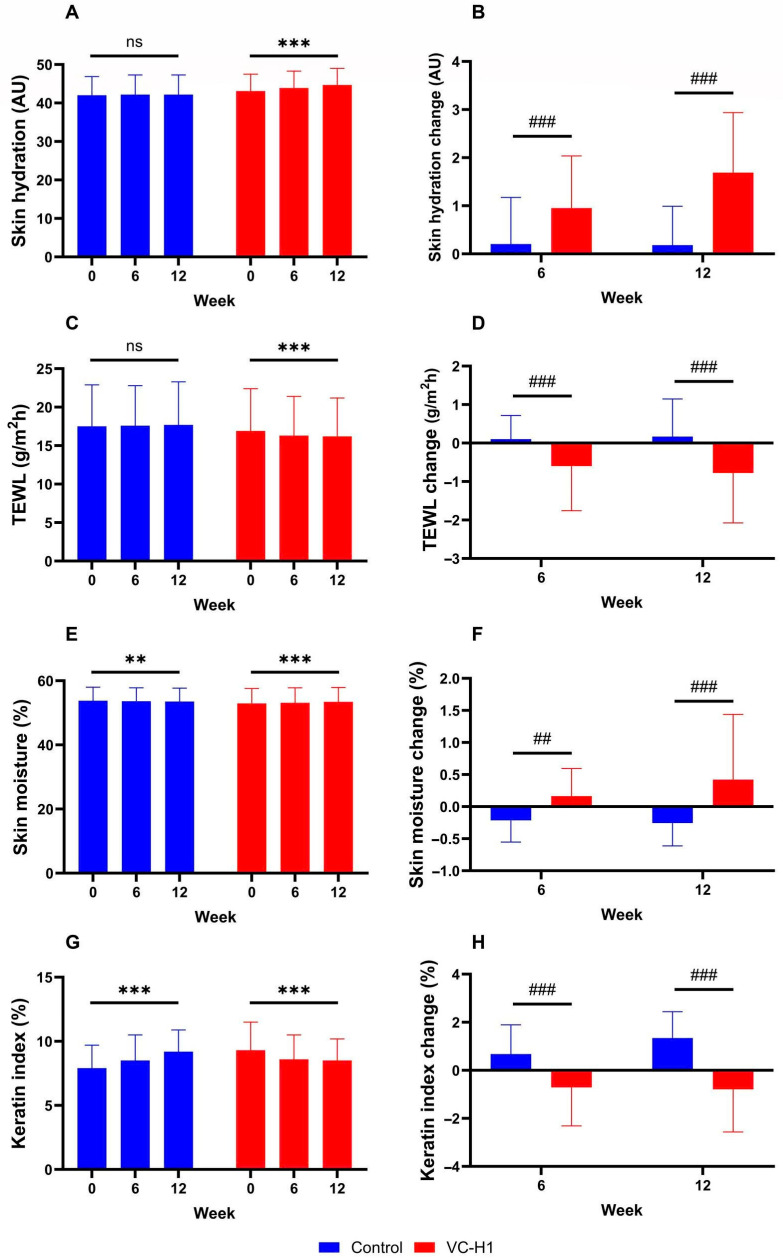
The effect of oral VC-H1 consumption on skin hydration (**A**,**B**), TEWL (**C**,**D**), skin moisture (**E**,**F**), and keratin index (**G**,**H**) after 12 weeks. Changes within each group were analyzed using a linear mixed-effect model adjusted for baseline values of each biomarker (** *p* < 0.01, *** *p* < 0.001). Differences between groups at weeks 6 and 12 were analyzed using a linear mixed-effect model (## *p* < 0.01, and ### *p* < 0.001). ns, not significant.

In the VC-H1 group, skin hydration significantly increased over 12 weeks (baseline: 43.1 ± 4.4 AU; week 6: 43.9 ± 4.4 AU; week 12: 44.7 ± 4.3 AU; *p* < 0.001; Figure 2A), with significantly greater improvement than that in the control group (*p* < 0.001; Figure 2B). TEWL significantly decreased in the VC-H1 group (baseline: 16.9 ± 5.5 AU; week 12: 16.2 ± 5.0 AU; *p* < 0.001; Figure 2C), with greater reductions than those in the control group at both time points (*p* < 0.001; Figure 2D). Skin moisture also significantly improved in the VC-H1 group (baseline: 52.9 ± 4.7 AU; week 12: 53.4 ± 4.5 AU; *p* < 0.001; Figure 2E), and the increase was superior to that in the control group (*p* < 0.001; Figure 2F). The keratin index decreased significantly in the VC-H1 group (baseline: 9.3 ± 2.2 AU; week 12: 8.5 ± 1.7 AU; *p* < 0.001; Figure 2G), whereas an opposite trend was observed in the control group (*p* < 0.001; Figure 2H). Overall, VC-H1 supplementation resulted in significant improvements in skin hydration, moisture, and keratin index, along with a significant reduction in TEWL compared with that observed for the control (*p* < 0.001).

### 2.3. Skin Elasticity

**Figure 3:**
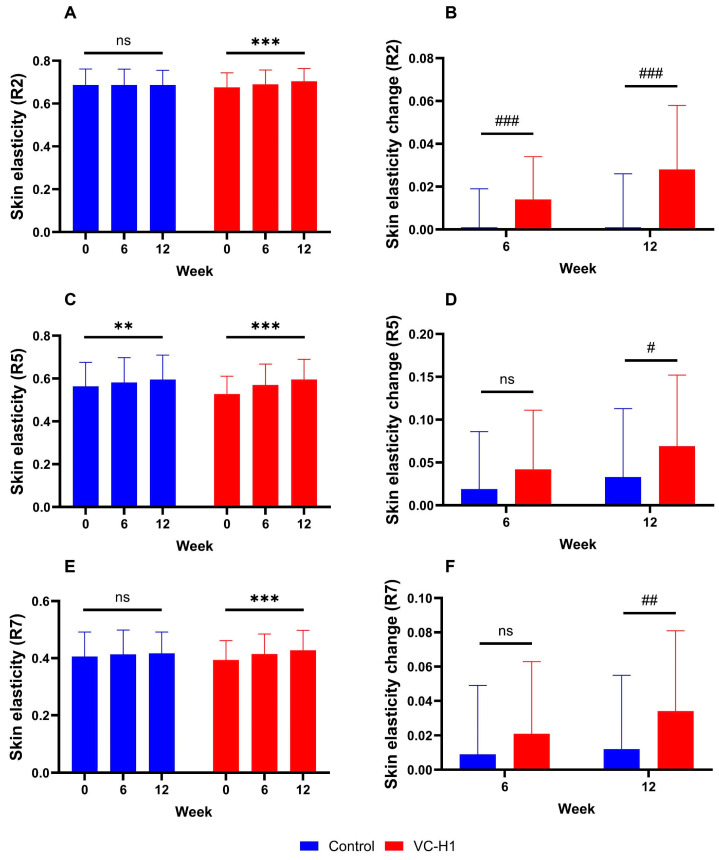
The effect of oral VC-H1 consumption on skin elasticity, as measured by R2 (**A**,**B**), R5 (**C**,**D**), and R7 (**E**,**F**) values after 12 weeks. Changes within each group over 12 weeks were analyzed using a linear mixed-effect model adjusted with the baseline value of each biomarker (** *p* < 0.01, *** *p* < 0.001). Differences between groups at weeks 6 and 12 were analyzed using a linear mixed-effects model (# *p* < 0.05, ## *p* < 0.01, and ### *p* < 0.001). ns, not significant.

Significant increases in R2, R5, and R7 values were observed in the VC-H1 group for 12 weeks (all *p* < 0.001; Figure 3A,C,E). These improvements were significantly greater than those in the control group at weeks 6 and 12 (*p* < 0.05; Figure 3B,D,F). In particular, R2 increased from 0.676 ± 0.068 AU at baseline to 0.704 ± 0.060 AU at week 12; R5 increased from 0.528 ± 0.083 AU to 0.596 ± 0.094 AU; and R7 increased from 0.394 ± 0.068 AU to 0.428 ± 0.070 AU. Overall, the VC-H1 group showed significant elasticity enhancement compared with that observed in the control group (*p* < 0.05).

### 2.4. Skin Texture

**Figure 4:**
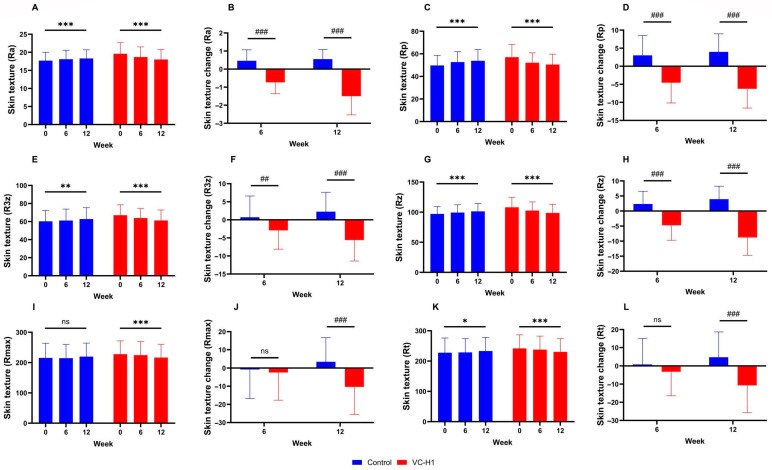
The effect of oral VC-H1 consumption on skin texture Ra (**A**,**B**), Rp (**C**,**D**), R3z (**E**,**F**), Rz (**G**,**H**), Rmax (**I**,**J**), and Rt (**K**,**L**) values after 12 weeks. Changes within each group over 12 weeks were analyzed using a linear mixed-effect model adjusted for baseline values of each biomarker (* *p* < 0.05, ** *p* < 0.01, *** *p* < 0.001). Differences between groups at weeks 6 and 12 were analyzed using a linear mixed-effect model (## *p* < 0.01, and ### *p* < 0.001). ns, not significant.

All texture roughness parameters (Ra, Rp, R3z, Rz, Rmax, and Rt) significantly reduced in the VC-H1 group over the 12-week period, indicating smoother skin (all *p* < 0.001; Figure 4A,C,E,G,I,K). These reductions were significantly greater than those observed in the control group (*p* < 0.05; Figure 4B,D,F,G,J,L). Notably, the control group showed an increasing trend in several parameters, contrasting with the consistent reduction observed in the VC-H1 group. Significant group-by-week interaction effects were used to confirm that these improvements were attributable to VC-H1 supplementation (*p* < 0.001).

### 2.5. Periorbital Wrinkles

**Figure 5:**
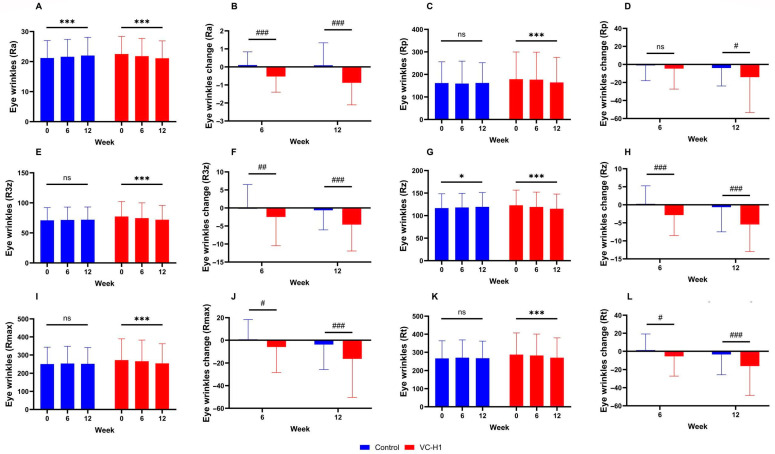
The effect of oral VC-H1 consumption on periorbital wrinkles Ra (**A**,**B**), Rp (**C**,**D**), R3z (**E**,**F**), Rz (**G**,**H**), Rmax (**I**,**J**), and Rt (**K**,**L**) values after 12 weeks. Changes within each group over 12 weeks were analyzed using a linear mixed-effects model adjusted for baseline values of each biomarker (* *p* < 0.05, *** *p* < 0.001). Differences between groups at weeks 6 and 12 were analyzed using a linear mixed-effects model (# *p* < 0.05, ## *p* < 0.01, and ### *p* < 0.001). ns, not significant.

Significant reductions in wrinkle parameters (Ra, Rp, R3z, Rz, Rmax, and Rt) were also observed in the VC-H1 group over 12 weeks (all *p* < 0.001; Figure 5A,C,E,G,I,K). These reductions were significantly greater than those in the control group, which, in contrast, showed stable or increasing values over time (*p* < 0.05; Figure 5B,D,F,H,J,L). The Ra and Rmax reductions were particularly prominent. Significant group-by-week interaction effects were observed for all wrinkle parameters (*p* < 0.001), highlighting the efficacy of VC-H1 in reducing periorbital wrinkles.

**Figure 6:**
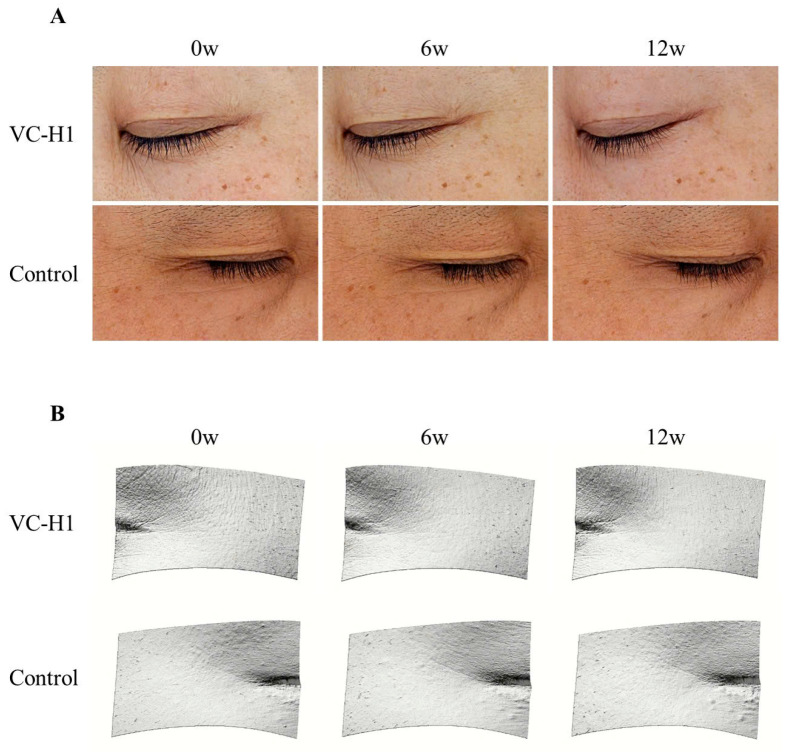
Visualization of periorbital wrinkle improvements in the VC-H1 and control groups over 12 weeks. (**A**) Representative clinical photographs (Mark-Vu^®^) at 0, 6, and 12 weeks (0w, 6w, 12w) show greater wrinkle reduction in the VC-H1 group compared to control. (**B**) 3D skin surface images (PRIMOS CR) from the same time points reveal visibly smoother texture and reduced wrinkle depth in the VC-H1 group relative to control.

Visual changes in periorbital wrinkles are presented in Figure 6. As illustrated, oral consumption of VC-H1 led to a more pronounced reduction in periorbital wrinkles in the treatment group compared to the control group.

### 2.6. Investigator-Assessed Overall Improvement

**Figure 7:**
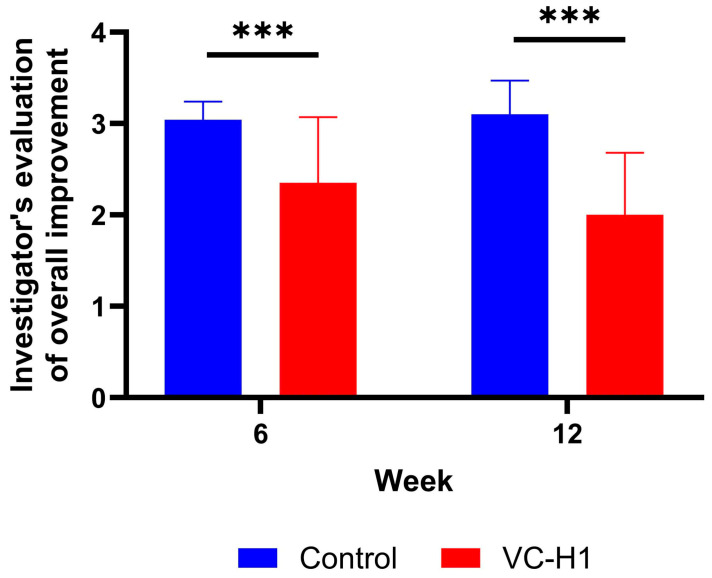
Evaluation of overall improvement by investigators. Linear mixed-effect model was used to compare the difference between the groups at week 6 and week 12, *** *p* < 0.001.

Investigator assessments of overall skin improvement showed significantly better scores in the VC-H1 group than those in the control group starting from week 6 and sustained through week 12 (VC-H1 group: 2.35 ± 0.72 at week 6, 2.00 ± 0.68 at week 12; control group: 3.04 ± 0.20 at week 6, 3.10 ± 0.37 at week 12; *p* < 0.001; Figure 7).

## 3. Discussion

*H. sabdariffa* has long been recognized for its medicinal properties, including its cardiovascular and metabolic benefits, as well as its antioxidant, anti-inflammatory, and anti-aging effects in dermatology [18]. In this randomized, placebo-controlled trial, oral supplementation with VC-H1 resulted in significant improvements in key skin parameters comprising increased hydration and elasticity, reduced TEWL, improved desquamation, and decreased wrinkle depth. Additionally, VC-H1 demonstrated a good safety profile, with no clinically significant abnormalities observed in hematology, clinical chemistry, or urinalysis.

Skin aging is closely associated with diminished hydration due to reactive oxygen species-induced degradation of hyaluronic acid and impairment of the skin barrier [22,23,24]. Photoaging further accelerates this process by disrupting lipid integrity, increasing TEWL, and causing irregular desquamation [25,26,27,28]. In our study, daily VC-H1 supplementation significantly improved hydration and skin barrier function. Reductions in TEWL and desquamation were observed from 6 weeks and sustained for 12 weeks. These findings are consistent with those of previous studies demonstrating hydration benefits as a result of *H. sabdariffa*-based interventions [19,21]. Such effects are possibly mediated by the abundant polyphenols, anthocyanins, and organic acids in VC-H1, which counter oxidative stress and stimulate hyaluronic acid synthesis by restoring fibroblast function and reducing degradative enzymes [13,18,19].

Declining skin elasticity and wrinkle formation are characteristic of aging skin, driven by oxidative damage, extracellular matrix (ECM) degradation, and elevated MMP activity [1,3,22,29,30]. Our findings showed that VC-H1 supplementation significantly enhanced skin elasticity and reduced wrinkle depth and surface roughness. As corroboration, in vitro evidence has shown that *H. sabdariffa* inhibits MMPs and collagenase while supporting collagen and elastin maintenance [13,15,16]. Additionally, an in vivo mouse model also demonstrated significant reductions in wrinkle depth and skin roughness as well as enhanced collagen production after administration of VC-H1 [19]. The ample amount of free amino acids contained in VC-H1 could be the vital element that facilitates the rejuvenative effects observed in these findings [20].

These effects are comparable to those reported for other botanical extracts with dermatologic benefits. Plant-based ingredients, such as *Cannabis sativa* and *Aerva lanata,* have been shown to improve skin hydration and inhibit elastase activity [31,32,33], while *Punica granatum* and *Olea europaea* protect collagen and promote ECM regeneration [34,35]. Furthermore, VC-H1 may present advantages over animal-derived collagen supplements, which, although effective in improving skin hydration and antioxidant defenses [7,36,37,38,39,40,41,42], can be associated with concerns about taste, allergenicity, and contamination risks [6,8,9,10]. Moreover, VC-H1 represents a promising, plant-based alternative for skin rejuvenation because of its favorable safety profile, pleasant formulation, and potent antioxidant properties.

The strengths of this study include its randomized, double-blind, placebo-controlled design and the use of objective, quantitative assessments of skin biophysical parameters, as well as the control of lifestyle and dietary factors. Furthermore, participant compliance was high, and dietary and physical activity levels were monitored throughout the study, supporting the reliability of the results. However, the 12-week duration may not capture long-term effects, and while the sample size was adequate for statistical analysis, larger, more diverse populations are needed to confirm these findings. In addition, the age range of our participants excluded those over 60 years old, who may exhibit more advanced photoaging. This limitation restricts the generalizability of VC-H1 findings to older age groups. The fact that all participants were of Korean ethnicity, with relatively uniform skin phototypes, also affects the applicability of our results. In future studies, long-term safety, optimal dosing, potential synergistic effects, and efficacy in other age groups, ethnicities, or skin types should be investigated.

## 4. Materials and Methods

### 4.1. Test Products

The test product was a liquid dietary supplement containing VC-H1, derived from the calyx of *H. sabdariffa* (Rawga Inc., Seongnam, Republic of Korea). It was classified as a functional food that promotes skin health and was administered once daily at a dose of 1.5 g, diluted in water. The placebo was a liquid formulation primarily composed of maltodextrin (without VC-H1), matched in appearance, taste, and weight to the test product to ensure the integrity of double blinding. The detailed compositions of both formulations are provided in Table S4.

### 4.2. Study Design and Participants

This randomized, double-blind, placebo-controlled clinical trial was conducted between March and September 2024. In total, 100 adults aged 35–60 years with dry skin and visible periorbital wrinkles (grade ≥ 3 on the visual wrinkle assessment scale; see Table S5) were enrolled. This age range typically exhibits early to moderate signs of photoaging, allowing us to detect measurable improvements in these parameters within a 12-week period, while minimizing the variability introduced by more advanced intrinsic aging processes commonly seen in older individuals. Participants were recruited through in-house posters and online advertising. The exclusion criteria are described in Table S6. Eligible participants were randomly assigned in a 1:1 ratio to either the VC-H1 (1.5 g/day) or control group, using computer-generated block randomization stratified by gender. The study period was 12 weeks, comprising four visits: screening (Week −2), randomization (Week 0), interim assessment (Week 6), and study completion (Week 12). Only participants with a skin hydration index of ≤49, as measured by the Corneometer^®^ CM 825 (Courage & Khazaka, Cologne, Germany), were included to ensure appropriate baseline conditions. To achieve adequate statistical power and account for an anticipated 20% dropout rate, 50 participants were allocated to each group. Group assignments were managed by an independent third party and remained sealed until the conclusion of the study, maintaining blinding for both participants and investigators. All participants were informed about the study’s objectives, procedures, and potential risks, and written informed consent was obtained before they participated. Trial registration: Clinical Research Information Service (cris.nih.go.kr). Identifier: KCT0009307.

### 4.3. Baseline Assessments

Comprehensive baseline assessments were conducted at the screening visit (Week −2), including demographic information, medical history, current medications, skin characteristics, cosmetic usage habits, lifestyle factors, and vital signs. Skin hydration, periorbital wrinkle assessment, and pregnancy testing (for women with childbearing potential) were performed. Blood and urine were analyzed at screening and at Week 12 to ensure participant eligibility and monitor safety (Tables S7–S10). Physical measurements were recorded at baseline and at the final visit. Participants were instructed to maintain their usual diet and lifestyle throughout the study period.

### 4.4. Skin Measurements

Efficacy assessments were conducted at Weeks 0, 6, and 12. The measurement site on the lateral face was selected based on the lowest hydration value recorded at the initial assessment. Before each measurement, participants rested for 30 min in a controlled environment (temperature 22–24 °C, humidity 45–55%) and refrained from drinking fluids for at least 1 h. Each parameter was measured in triplicate, with the average value used for analysis.

- Skin hydration was measured using the Corneometer^®^ CM 825 (Courage & Khazaka, Cologne, Germany), which is used to assess skin capacitance to quantify moisture content in the stratum corneum (depth of 10–20 μm). The results are presented as AU, which is a relative measurement scale used to express values derived from instrument-based readings with no existing absolute physical unit. This type of unit allows for consistent comparison across time points or between groups within the same experimental setting.
- TEWL was evaluated using the Vapometer^®^ (Delfin Technologies Ltd., Kuopio, Finland), measuring water evaporation at the eye corner and nose tip by monitoring changes in chamber humidity.
- Deep skin moisture content was assessed using the Moisturemeter D Compact (Delfin Technologies Ltd., Kuopio, Finland), which is used to measure water content (PWC%) at a depth of 2–2.5 mm with a 265 MHz electromagnetic wave.
- The Keratin index was measured by collecting keratinocytes with a D-squame Standard Sampling Disc, followed by an analysis using the Visioscan^®^ VC98 (Courage & Khazaka, Cologne, Germany). The desquamation index (D.I., %) was calculated, with lower values indicating improved desquamation.
- Skin elasticity was measured using the Cutometer^®^ MPA580 (Courage & Khazaka, Cologne, Germany) at a suction pressure of 450 mbar (2.0 s on/off cycles), with R2, R5, and R7 values recorded. Increases in these parameters (approaching 1) indicate an enhanced elasticity (Table S11).
- Skin texture was assessed using the PRIMOS CR (GF Messtechnik, Teltow, Germany), a three-dimensional (3D) optical measurement device that is used to evaluate roughness parameters (Ra, Rp, R3z, Rz, Rmax, Rt) at the eye corner and nose tip junction (Table S12).
- Periorbital wrinkles were evaluated visually using the Mark-Vu^®^ (PSIPLUS, Suwon, Republic of Korea) and quantitatively via 3D analysis with the PRIMOS CR (GF Messtechnik, Teltow, Germany), using the same roughness parameters (Table S12). Lower values reflected improvements in skin quality.
- Overall skin condition improvement was assessed following pre-determined criteria agreed upon by the investigators (Table S13).

### 4.5. Safety and Compliance Assessments

Test products and placebos were dispensed during the second and third visits, and the remaining products were collected at the third and fourth visits to assess compliance. Compliance was considered satisfactory if participants consumed >80% of the assigned treatment, assessed by the number of remaining products collected at the third and fourth visits. Participants were instructed to report any adverse events, which were monitored through clinical assessments during each visit. Adverse events included the occurrence of fever, symptoms, or significant abnormalities detected in laboratory tests.

### 4.6. Statistical Analysis

Continuous variables are expressed as means ± standard deviations, whereas frequencies are used for categorical variables. Data normalization was performed where necessary. Baseline characteristics were compared using two-sample *t*-tests (for continuous variables) and Chi-square or Fisher’s exact tests (for categorical variables). Dietary intake, physical activity, and efficacy outcomes were analyzed using linear mixed-effects models, with group, time, and their interaction as fixed effects. Random effects were used to account for individual variability. Baseline values were included as covariates to reduce confounding bias. Safety data were analyzed using linear mixed-effects models for continuous variables and Fisher’s exact or McNemar’s tests for categorical variables. As the objective of this study was to comprehensively evaluate a range of relevant physiological biomarkers rather than confirm efficacy based on a single primary endpoint, no primary or secondary endpoints were predefined. Therefore, correction for multiple comparisons was not applied to avoid overly conservative corrections, which may increase the risk of type II error and mask meaningful physiological changes. All statistical analyses were conducted using SAS^®^ version 9.4, with statistical significance set at *p* < 0.05.

## 5. Conclusions

Our findings show that oral supplementation with VC-H1 significantly improves skin hydration, reduces TEWL, enhances elasticity, and decreases desquamation and wrinkle depth in photoaged individuals over a 12-week period. These clinical benefits are likely mediated by the rich content of anthocyanins, polyphenols, and organic acids, which exert potent antioxidant effects and inhibit matrix metalloproteinase activity in VC-H1, supporting ECM preservation and skin barrier function. Given its safety, favorable compliance, and efficacy, VC-H1 presents a promising plant-based alternative to animal-derived collagen supplements for skin rejuvenation. Nevertheless, future large-scale, long-term studies are needed to confirm these findings in a wider age range and other ethnicities, as well as determine optimal dosing regimens and further elucidate the molecular mechanisms underlying its skin benefits.

## Data Availability

The raw data supporting the conclusions of this article will be made available by the authors on request.

## Supplementary Materials

The following supporting information can be downloaded at https://www.mdpi.com/article/10.3390/ijms26157291/s1.

## Abbreviations

The following abbreviations are used in this manuscript:

MMPs	Matrix Metalloproteinases.
TEWL	Transepidermal Water Loss.
VC-H1	Hibiscus Enzyme Extract.
ECM	Extracellular Matrix.
Ra	Roughness average of the roughness profile.
Rt	The distance between the highest and the lowest point of the roughness profile.
Rp	Maximum peak height of the roughness profile.
R3z	The third-point height of the roughness profile.
Rmax	Ten-point height of the roughness profile.
