# Correction: Baek et al. Hibiscus Collagen Alternative (VC-H1) as an Oral Skin Rejuvenating Agent: A 12-Week Pilot Study. *Int. J. Mol. Sci.* 2025, 26, 7291

**DOI:** 10.3390/ijms262010115

**Published:** 2025-10-17

**Authors:** Yujin Baek, Ngoc Ha Nguyen, Young In Lee, Min Joo Jung, In Ah Kim, Sung Jun Lee, Hyun Min Kim, Ju Hee Lee

**Affiliations:** 1 Department of Dermatology & Cutaneous Biology Research Institute, Yonsei University College of Medicine, Seoul 03722, Republic of Korea; uj200076@yuhs.ac (Y.B.); ngocha7996@yuhs.ac (N.H.N.); ylee1124@yuhs.ac (Y.I.L.); 2 Department of Dermatology, University of Medicine and Pharmacy at Ho Chi Minh City, Ho Chi Minh City 17000, Vietnam; 3 Scar Laser and Plastic Surgery Center, Yonsei Cancer Hospital, Seoul 03722, Republic of Korea; 4 Global Medical Research Center Co., Ltd., Seoul 06526, Republic of Korea; mjung11@gmrc.co.kr (M.J.J.); inah@gmrc.co.kr (I.A.K.); 5 Liting Plastic Surgery, Seoul 06035, Republic of Korea; justdisj@naver.com; 6 Rawga Inc., Seongnam-si 13590, Republic of Korea; raw@rawga.com

In the original publication [1], there was a mistake in Table S4b as published. During a post-publication verification of our manuscript, we discovered that the amino acid composition table included in the published manuscript was inadvertently transcribed from an earlier version of the analysis data. We have prepared a corrected table based on the latest verified data and are submitting it here for inclusion as a minor correction. The corrected Table S4b appears below. The authors state that the scientific conclusions are unaffected. This correction was approved by the Academic Editor. The original publication has also been updated.

**Table S4b:** Chemical composition of the test product.

Sample name	Analyzed components	Measured values (%)
Hibiscus Enzyme Extract	Asp	1.44 (%)
	Glu	0.44 (%)
	Ser	0.14 (%)
	His	0.14 (%)
	Gly	19.11 (%)
	Thr	0.11 (%)
	Arg	0.22 (%)
	Ala	0.23 (%)
	Tyr	0.05 (%)
	Cys	ND
	Val	0.11 (%)
	Met	ND
	Trp	0.04 (%)
	Phe	0.10 (%)
	Ile	0.06 (%)
	Leu	0.14 (%)
	Lys	0.13 (%)
	Hypro	9.74 (%)
	Pro	6.89 (%)

Total anthocyanin was analyzed using Ultraviolet-visible spectrophotometry, and amino acid composition was analyzed using High-Performance Liquid Chromatography with Fluorescence Detection. ND (Not Detected) indicates that the component was below the quantification limit; the quantification limit represents the detectable result, with amino acid composition being 0.1%.
