# Supplementary material for: Hibiscus Collagen Alternative (VC-H1) as an Oral Skin Rejuvenating Agent: A 12-Week Pilot Study
Source: Int J Mol Sci. 2025 Jul 28;26(15):7291. doi: 10.3390/ijms26157291 (PMC12347160; doi:10.3390/ijms26157291)
Supplement: Supplementary file 1 [file ijms-26-07291-s001.zip › ijms-3688752-supplementary.pdf]

Table S1. Baseline characteristics

Variables	Control	VC-H1	p*
Age (years, mean ± SD)	44.6 ± 6.9	43.4 ± 6.0	0.347
Gender (male/female)	22 / 28	21 / 29	0.840
Menstruation (Y/N/NA)	5 / 23 / 22	4 / 25 / 21	0.915
Alcohol amount (standard drink/week, mean ± SD)	1.6 ± 3.4	1.3 ± 2.5	0.570
Recommended Food Score (mean ± SD)	23.0 ± 9.7	20.1 ± 9.9	0.142
Skin type (dry/neutral/oily/combination/acne)	21 / 12 / 3 / 14 / 0	22 / 11 / 4 / 13 / 0	1
Skin hydration (AU, mean ± SD)	42.0 ± 4.9	43.1 ± 0.6	0.265
Eye wrinkles (grade, mean ± SD)	4.3 ± 0.9	4.3 ± 0.9	0.733

NA, not applicable; SD, standard drink; AU, arbitrary unit.

*Two-sample t-test for continuous variables and Chi-square or Fisher's exact test for categ orical variables were used to compare the differences between the groups

Table S2. Compliance

Week	Compliance (mean ± SD)		p*
	Control	VC-H1	
Week 6	98.9 ± 0.9	97.7 ± 1.3	0.440
Week 12	99.8 ± 1.0	100.1 ± 1.0	0.854
Total	99.3 ± 0.7	98.8 ± 0.9	0.626

*Two-sample t-test

Table S3. Dietary intake and physical activity

Variables	Control	VC-H1	p*		
			Group	Week	Group * Week
Dietary intake (mean ± SD)					
Energy (kcal/d)					
Week 0	1470 ± 45	1447 ±36			
Week 6	1464 ± 39	1514 ± 48			
Week 12	1468 ± 45	1560 ± 46	0.508	0.117	0.120
p**	0.977	0.004			
Carbohy-drate(g/d)					
Week 0	205 ± 6	206 ± 6			
Week 6	208 ± 6	210 ± 8			
Week 12	212 ± 7	218 ± 8	0.750	0.103	0.918
p**	0.219	0.078			
Fat (g/d)					
Week 0	46 ± 2	45 ± 2			
Week 6	45 ± 2	48 ± 2			
Week 12	44 ± 2	49 ± 2	0.335	0.770	0.068
p**	0.237	0.052			
Sodium (mg/d)					
Week 0	3003 ± 141	3018 ± 147			
Week 6	3110 ± 155	3054 ± 152			
Week 12	3091 ± 143	3313 ±152	0.772	0.123	0.293
p**	0.501	0.029			
Physical activity (MET-min/week, mean ± SD)					
Week 0	3641 ± 409	3080 ± 335			
Week 6	3137 ± 329	2413 ± 274			
Week 12	3226 ± 392	2729 ± 291	0.133	0.041	0.861
p**	0.193	0.277			

MET, metabolic equivalent task

*Linear mixed-effect model was used to analyze the effects of group, week and gro up*week for 12 weeks

**Linear mixed-effect model was used to analyze the changes within each group for 12 weeks

Table S4a. The ingredients of the test and control products.

Test product (20 g)		Control product (20 g)	
Ingredient	Ratio (%)	Ingredient	Ratio (%)
30% Hibiscus enzyme extract	25.000	Maltodextrin	25.000
Purified Water	55.860	Purified Water	56.528
Fructose	18.000	Fructose	18.000
Sodium Bicarbonate	0.900	Citric Acid	0.150
Sodium Citrate	0.200	Pomegranate Flavor	0.200
Sucralose	0.040	Xanthan Gum	0.100
		FD&C Red No. 40	0.020
		FD&C Blue No. 1	0.002
Total	100.000	Total	100.000

Table S4b. Chemical composition of the test product

Sample name	Analyzed components	Measured values (%)
Hibiscus Enzyme Extract	Asp	1.44 (%)
	Glu	0.44 (%)
	Ser	0.14 (%)
	His	0.14 (%)
	Gly	19.11 (%)
	Thr	0.11 (%)
	Arg	0.22 (%)
	Ala	0.23 (%)
	Tyr	0.05 (%)
	Cys	ND
	Val	0.11 (%)
	Met	ND
	Trp	0.04 (%)
	Phe	0.10 (%)
	Ile	0.06 (%)
	Leu	0.14 (%)
	Lys	0.13 (%)
	Hypro	9.74 (%)
	Pro	6.89 (%)

Total anthocyanin was analyzed using Ultraviolet-visible spectrophotometry, and amino acid composition was analyzed using High-Performance Liquid Chromatography with Fluorescence Detection. ND (Not Detected) indicates that the component was below the quantification limit; the quantification limit represents the detectable result, with amino acid composition being 0.1%.

Table S5. Visual Assessment Scale

Grade	Description
0	No wrinkles
1	Very shallow or lines, yet visible wrinkles
2	Just visible wrinkles, like a hazy crease
3	Visible wrinkles, like light, clear creases
4	Clearly visible wrinkle
5	Clearly visible wrinkles and well-defined edges
6	Moderately deep wrinkle
7	Deep wrinkles and carved edges
8	The deep and prominent wrinkle with furrow
9	Redundant folds

Table S6. Exclusion Criteria

Exclusion Criteria
● Presence of skin abnormalities such as moles, acne, erythema, or telangiectasia at the measurement site<br>● Current diagnosis or treatment for skin diseases such as atopic dermatitis or psoriasis<br>● Use of oral contraceptives, hormone therapy, diuretics, or immunosuppressants<br>● Use of systemic or topical products containing steroids or retinoids within 3 months prior to the first visit<br>● Use of anti-wrinkle cosmetic products including retinoids, retinol, AHA, or moisture-rich products within 2 weeks prior to the first visit<br>● Receipt of dermatologic procedures such as skin resurfacing, laser treatments, chemical peeling, or other facial treatments within 1 month, or procedures such as Botox or fillers on the face within 6 months prior to the first visit<br>● Individuals with any of the following conditions:<br>• Uncontrolled hypertension (SBP ≥160 mmHg or DBP ≥100 mmHg) or hypotension (SBP <90 mmHg or DBP <60 mmHg)<br>• Uncontrolled diabetes mellitus (fasting blood glucose ≥180 mg/dL)<br>• Renal dysfunction (serum creatinine ≥2.4 mg/dL)<br>• Hepatic dysfunction (AST or ALT ≥120 U/L)<br>• Mental disorders including schizophrenia, depression, drug or alcohol addiction<br>• Hospitalization or treatment for skin diseases, asthma, heart disease, or central nervous system disorders<br>● Continuous intake of herbal medicines, dietary supplements, or products containing Hibiscus or collagen within 1 month prior to the first visit<br>● Current smokers or individuals who have quit smoking within the past year<br>● Participation in other clinical trials within 1 month prior to the first visit (within 6 months for skin-related trials), or plans to participate in another clinical study during the study period<br>● Pregnant or breastfeeding women, or individuals planning pregnancy during the study period<br>● History of hypersensitivity to ingredients in the test or control products, or experience of severe allergic reactions to food<br>● Any individual deemed unsuitable for participation by the investigator

Table S7. Hematological test

Variables (mean ± SD)	Control	VC-H1	p*		
			Group	Week	Group * Week
WBC ($10^3/\mu\ell$)					
Week 0	5.4 ± 1.4	5.6 ± 1.6			
Week 12	5.6 ± 1.2	5.5 ± 1.3	0.827	0.918	0.361
p**	0.472	0.566			
RBC ($10^6/\mu\ell$)					
Week 0	4.5 ± 0.4	4.6 ± 0.4			
Week 12	4.6 ± 0.4	4.5 ± 0.5	0.996	0.775	0.205
p**	0.485	0.272			
Hb (g/dℓ)					
Week 0	13.8 ± 1.3	13.8 ± 1.4			
Week 12	13.9 ± 1.3	13.7 ± 1.5	0.654	0.959	0.239
p**	0.384	0.425			
Hct (%)					
Week 0	41.9 ± 3.5	41.9 ± 3.8			
Week 12	41.6 ± 3.3	41.1 ± 3.9	0.711	0.004	0.221
p**	0.232	0.004			
PLT (Thous/$\mu\ell$)					
Week 0	257.8 ± 55.2	266.2 ± 50.8			
Week 12	269.7 ± 56.9	266.8 ± 47.7	0.778	0.035	0.038
p**	0.003	0.978			
Neutrophil (%)					
Week 0	55.8 ± 8.2	54.9 ± 8.8			
Week 12	55.1 ± 8.6	52.6 ± 8.7	0.300	0.090	0.383
p**	0.556	0.070			
Lymphocytes (%)					
Week 0	34.1 ± 7.5	34.6 ± 7.9			
Week 12	34.2 ± 7.8	36.2 ± 8.3	0.439	0.295	0.338
p**	0.948	0.157			
Monocytes (%)					
Week 0	7.0 ± 1.7	6.9 ± 1.9			
Week 12	7.2 ± 2.0	7.1 ± 1.9	0.850	0.306	0.991
p**	0.474	0.464			
Eosinophils (%)					
Week 0	2.4 ± 1.6	2.9 ± 2.2			
Week 12	2.9 ± 1.9	3.4 ± 2.8	0.192	0.013	0.959
p**	0.070	0.082			
Basophils (%)					
Week 0	0.7 ± 0.4	0.7 ± 0.4			
Week 12	0.7 ± 0.4	0.8 ± 0.4	0.831	0.425	0.255

p**	0.808	0.172

WBC, white blood cell; RBC, red blood cell; Hb, hemoglobin; Hct, hematocrit; PLT, platelet.

*Linear mixed-effect model was used to analyze the effects of group, week and group*week for 12 weeks

**Linear mixed-effect model was used to analyze the changes within each group for 12 weeks

Table S8. Blood chemistry test

Variables (mean ± SD)	Control	VC-H1	p* Group	p* Week	p* Group * Week
T.Protein (g/dℓ)					
Week 0	6.9 ± 0.3	7.0 ± 0.4			
Week 12	6.9 ± 0.4	6.9 ± 0.3	0.781	0.668	0.084
p**	0.355	0.127			
Albumin (g/dℓ)					
Week 0	4.3 ± 0.2	4.3 ± 0.2			
Week 12	4.3 ± 0.2	4.3 ± 0.2	0.377	0.400	0.709
p**	0.390	0.739			
BUN (mg/dℓ)					
Week 0	12.6 ± 2.9	13.6 ± 3.6			
Week 12	12.6 ± 3.0	12.9 ± 3.1	0.252	0.251	0.234
p**	0.976	0.100			
Creatinine (mg/dℓ)					
Week 0	0.7 ± 0.2	0.8 ± 0.2			
Week 12	0.7 ± 0.2	0.8 ± 0.2	0.616	0.530	0.804
p**	0.536	0.788			
T. bilirubin (mg/dℓ)					
Week 0	0.8 ± 0.2	0.7 ± 0.2			
Week 12	0.7 ± 0.2	0.7 ± 0.3	0.665	0.347	0.161
p**	0.099	0.743			
AST (U/L)					
Week 0	22.7 ± 6.0	21.8 ± 4.8			
Week 12	22.7 ± 6.6	21.2 ± 4.7	0.265	0.484	0.571
p**	0.925	0.371			
ALT (U/L)					
Week 0	21.0 ± 12.2	20.2 ± 10.6			
Week 12	20.2 ± 11.6	20.4 ± 10.7	0.823	0.483	0.694
p**	0.439	0.827			
GGT (U/L)					
Week 0	23.5 ± 16.5	23.4 ± 16.6			
Week 12	22.9 ± 12.5	24.8 ± 20.2	0.767	0.802	0.334
p**	0.612	0.390			
Glucose (mg/dℓ)					
Week 0	89.8 ± 8.4	87.0 ± 6.3			
Week 12	87.3 ± 8.5	86.6± 6 .8	0.224	0.013	0.085
p**	0.003	0.573			

T.protein, total protein; BUN, blood urea nitrogen; T.bilirubin, total bilirubin; AST, a spartate aminotransferase; ALT, alanine aminotransferase; GGT, gamma glutamyltran sferase.

*Linear mixed-effect model was used to analyze the effects of group, week and gro up*week for 12 weeks

**Linear mixed-effect model was used to analyze the changes within each group for 12 weeks

Table S9. Urine test

Variables (mean ± SD)	Control		VC-H1		p*
	NCS	CS	NCS	CS	
pH					
Week 0	50	0	50	0	-
Week 12	49	0	49	0	-
p**	-		-		
Nitrite					
Week 0	50	0	50	0	-
Week 12	49	0	49	0	-
p**	-		-		
Specific gravity					
Week 0	50	0	50	0	-
Week 12	49	0	49	0	-
p**	-		-		
Protein					
Week 0	50	0	50	0	-
Week 12	49	0	49	0	-
p**	-		-		
Glucose					
Week 0	50	0	50	0	-
Week 12	49	0	49	0	-
p**	-		-		
Ketone					
Week 0	50	0	50	0	-
Week 12	49	0	49	0	-
p**	-		-		
Bilirubin					
Week 0	50	0	50	0	-
Week 12	49	0	49	0	-
p**	-		-		
Occult blood					
Week 0	50	0	50	0	-
Week 12	49	0	49	0	-
p**	-		-		
Urobilinogen					
Week 0	50	0	50	0	-
Week 12	49	0	49	0	-
p**					
Leukocyte esterase					
Week 0	50	0	50	0	-
Week 12	49	0	49	0	-
p**	-		-		

Number of subjects. NCS, not clinically significant; CS, clinically significant.
Fishers' exact test was used to compare the difference between the groups and p-values were not computed because CS column contains all zeros.
McNemar's test was used to compare the difference within each group and p-values were not computed because CS column contains all zeros.

Table S10. Normal ranges for hematological test, blood chemistry test and urine test

Variables	Normal range
Hematological test	
WBC (10^3/㎕)	4.0–10.0
RBC (10^6/㎕)	M: 4.00-6.00 / F: 3.80-5.30
Hb (g/㎗)	M: 13.0-17.5 / F: 12.0-16.0
Hct (%)	M: 38.0-52.0 / F: 36.0-46.0
PLT (10^3/㎕)	150-450
Neutrophils (%)	40.8-80.0
Lymphocytes (%)	15.0-44.0
Monocytes (%)	2.0-10.0
Eosinophils (%)	0.0-5.0
Basophils (%)	0.0-3.0
Blood chemistry test	
T.protein (g/㎗)	6.6 - 8.7
Albumin (g/㎗)	3.5 - 5.2
BUN (㎎/㎗)	8 - 22
Creatinine (㎎/㎗)	M: 0.70 - 1.30 / F: 0.50 - 1.10
T.bilirubin (㎎/㎗)	≤ 1.2
AST (U/L)	< 40
ALT (U/L)	< 40
GGT (U/L)	M: 0.70 - 1.30 / F: 0.50 - 1.10
Glucose (㎎/㎗)	70-99
Urine test	
pH	4.8-7.4
Nitrite	Negative
Specific gravity	1.005-1.030
Protein	Negative
Glucose	Negative
Ketone	Negative
Bilirubin	Negative
Occult blood	Negative
Urobilinogen	Normal
Leukocyte esterase	Negative

Normal ranges are from Global Medical Research Center. WBC, white blood cell; RBC, red blood cell; Hb, hemoglobin; Hct, hematocrit; PLT, platelet; T.protein, total protein; BUN, blood urea nitrogen; T.bilirubin, total bilirubin; AST, aspartate aminotransferase; ALT, alanine aminotransferase; GGT, gamma glutamyltransferase.

Table S11. Description of skin elasticity parameters

Parameter		Description
R2	Ua/Uf	Total skin elasticity, including mucosal deformation (closer to 1 indicates higher elasticity)
R5	Ur/Ue	Total skin elasticity excluding mucosal deformation
R7	Ur/Uf	Reflects skin elasticity, independent of skin thickness

Ua: total retraction (mm), Ue: immediate distention (mm), Uf: total elongation (mm), Ur: immediate retraction (mm)

Table S12. Description of skin and periorbital wrinkle parameters

Parameter	Description
Ra	Roughness average of the roughness profile
Rp	Maximum peak height of the roughness profile
R3z	The third-point height of the roughness profile
Rz	Ten-point height of the roughness profile
Rmax	Maximum peak-to-valley roughness height of the roughness profile
Rt	The distance between the highest and the lowest point of the roughness profile

Table S13. Overall Improvement assessment by investigators

Score	Classification	Description
1	Very Good	Overall marked improvement of symptoms
2	Good	Overall improvement of symptoms
3	Unchanged	No difference
4	Bad	Overall worsening of symptoms
5	Very Bad	Overall marked worsening of symptoms
